# Psychological Flexibility as a Mediator Between Parenting Stress and Depression Among Chinese Primary Caregivers of Infants: A Cross-Sectional Study

**DOI:** 10.3390/nursrep16080278

**Published:** 2026-08-08

**Authors:** Jie Zhou, Shang Gao, Qi Jiang, Yiwei Qian, Wei Zhao, Wenxuan Ding, Lei Yang

**Affiliations:** 1 School of Nursing, Health Science Center, Xi’an Jiaotong University, Xi’an 710061, China; zhoujie0924@stu.xjtu.edu.cn (J.Z.); gaoshang2002@stu.xjtu.edu.cn (S.G.); 3124315116@stu.xjtu.edu.cn (W.Z.); dwx1123@stu.xjtu.edu.cn (W.D.); 2 School of Public Health, University of California, Berkeley, CA 94720, USA; qi_jiang@berkeley.edu; 3 Research Institute of Economics and Management, Southwestern University of Finance and Economics, Chengdu 611130, China; qianyw@swufe.edu.cn

**Keywords:** cross-sectional study, parenting stress, psychological flexibility, depression, caregiver, infant, nursing

## Abstract

**Background:** Early childhood caregivers are exposed to intensive caregiving demands and have a high risk of depression. Guided by the family stress process model and Acceptance and Commitment Therapy (ACT), this cross-sectional study examined whether psychological flexibility mediates the association between parenting stress and depression. **Methods:** A cross-sectional survey was conducted among 1140 Chinese primary caregivers of infants aged 0–36 months in Ya’an, Sichuan, China, from 9 October 2022 to 8 October 2025 using convenience sampling. Validated scales included the Parenting Stress Index-Short Form (PSI-SF), Acceptance and Action Questionnaire-II (AAQ-II) (Higher AAQ-II scores reflected lower psychological flexibility), and Patient Health Questionnaire-8 (PHQ-8). Hayes’ PROCESS Model 4, with 5000 bootstrap resamples, was adopted for the mediation analysis. **Results:** Parenting stress was positively associated with depressive symptoms (r = 0.335, *p* < 0.01) (weak-to-moderate effect size). Psychological flexibility exerted a partial mediating effect between the two variables, and the indirect effect accounted for 45% of the total effect. Caregivers with lower household income, lower educational level, and those caring for children aged 0–12 months reported significantly higher depression (all *p* < 0.05). **Conclusions:** Parenting stress is an important risk factor for depression, and psychological flexibility plays a significant partial mediating role in this relationship. ACT-based interventions targeting psychological flexibility may hold implications for depression among early childhood caregivers.

## 1. Introduction

Early childhood (0–36 months) constitutes a critical period for physical, cognitive, and socio-emotional development. During this stage, caregivers’ physical and mental health play a vital role in shaping infants’ attachment security, emotional regulation, and social adaptability, thereby exerting a lasting influence on developmental trajectories [1,2]. As the primary companions and caregivers during early development, individuals are responsible for intensive, continuous caregiving tasks—including feeding, daily care, and early stimulation. Beyond these physical demands, they must also navigate persistent sleep deprivation and the psychological challenges of transitioning into new parental roles [3,4]. This unique cluster of chronic, uncontrollable stressors creates a discrete high-risk mental health subgroup, which warrants targeted mechanistic research.

In recent years, depression in caregivers of early childhood has become a major public health issue of global concern, that is, a cross-cultural crisis [5,6,7,8]. According to prevalence data, the incidence of depression in Chinese infant and child caregivers is 15% to 30%, which far exceeds the baseline depression rate of the general adult population, with the highest incidence in the postpartum 3–6 months [9]. From a bidirectional developmental perspective, caregiver depression generates reciprocal harm: it impairs caregivers’ own psychological functioning, while disrupting sensitive parent–child interaction quality and increasing the likelihood of infant emotional and behavioral dysregulation [5,10,11]. In severe cases, depression may escalate to self-harm or suicidal behavior, threatening family functioning and social stability [11,12]. These findings underscore the urgent need to identify modifiable psychological pathways linking caregiving burden to depressive outcomes.

Parenting stress has been uniformly identified as a primary risk factor for depression among caregivers of early childhood [13,14,15]. It refers to persistent psychological strain arising when caregiving demands exceed available personal and social coping resources [16]. It originates from three overlapping domains of infant-related challenges: infant-specific behavioral challenges, structural work-family role conflict, and insufficient instrumental and emotional social support [17]. Chronic parenting stress triggers different physiological (elevated cortisol, postpartum neural structural plasticity), psychological (negative self-evaluation), and social (intergenerational family conflict) stress burdens [18,19,20]. These multi-layered strains jointly impair the brain emotion-regulatory circuitry and reduced capacity for adaptive acceptance of distressing caregiving experiences [21,22]. Stress and emotion regulation theories provide a foundational context for this association: chronic unmitigated caregiving stress progressively depletes limited self-regulatory psychological resources, accelerates the accumulation of negative affective states, and creates sustained vulnerability to clinical depression [6,23].

Derived from Acceptance and Commitment Therapy (ACT), psychological flexibility refers to the capacity to remain open to internal experiences while flexibly adjusting behavior in accordance with personally held values [24]. Growing evidence indicates that psychological flexibility plays a key mediating role in the relationship between stress and depression within the framework of Acceptance and Commitment Therapy (ACT). For example, a 2023 study showed that individuals with higher psychological flexibility are better able to tolerate distressing experiences and regulate negative emotions, thereby reducing depression [25]. Conversely, psychological inflexibility is characterized by experiential avoidance and cognitive rigidity, which intensify stress-related distress and increase the risk of depression [26,27].

In the context of infant caregiving, sustained parenting stress systematically erodes psychological flexibility through exposure to unmanageable caregiving demands [28]. Repeated exposure to uncontrollable caregiving stressors leads to decreased psychological flexibility, which further limits caregivers’ ability to cope with parenting stress, increases reliance on maladaptive coping strategies, and ultimately exacerbates depressive symptoms, forming a self-reinforcing cycle: parenting stress weakens psychological flexibility, and then aggravates depression [29,30]. Meanwhile, no prior work has integrated biopsychosocial, emotion regulation, and coping frameworks to systematically unpack this mediating pathway specifically for infant caregivers, a population facing distinct, age-specific chronic care burdens.

The core of Acceptance and Commitment Therapy (ACT) is to cultivate psychological flexibility to buffer stress and depression, which originated from the Western individualistic cultural context centered on personal value pursuit [31,32]. This may conflict with East Asian cultural norms that focus on family harmony and intergenerational care obligations [33]. Chinese infant and toddler care providers are under unique cultural pressures, including the conflict of multi-generation parenting, the pressure of social norms to seek the “perfect mother and child”, and the limited support of the state for infant and toddler care [34,35]. Although existing Western studies have confirmed that psychological flexibility mediates the stress–depression link, it is unclear whether this mechanistic pathway and ACT-based intervention logic have consistent predictive power among Chinese early childhood caregivers.

Therefore, the present study aims to examine the relationships among parenting stress, psychological flexibility, and depression among primary caregivers of children aged 0–36 months, with a particular focus on testing the mediating role of psychological flexibility. Based on existing theoretical frameworks and empirical findings, we propose the following hypotheses:

**H1.**  *Parenting stress is positively associated with depression among infant caregivers.*

**H2.**  *Psychological flexibility is negatively related to parenting stress and depression.*

**Figure 1:**
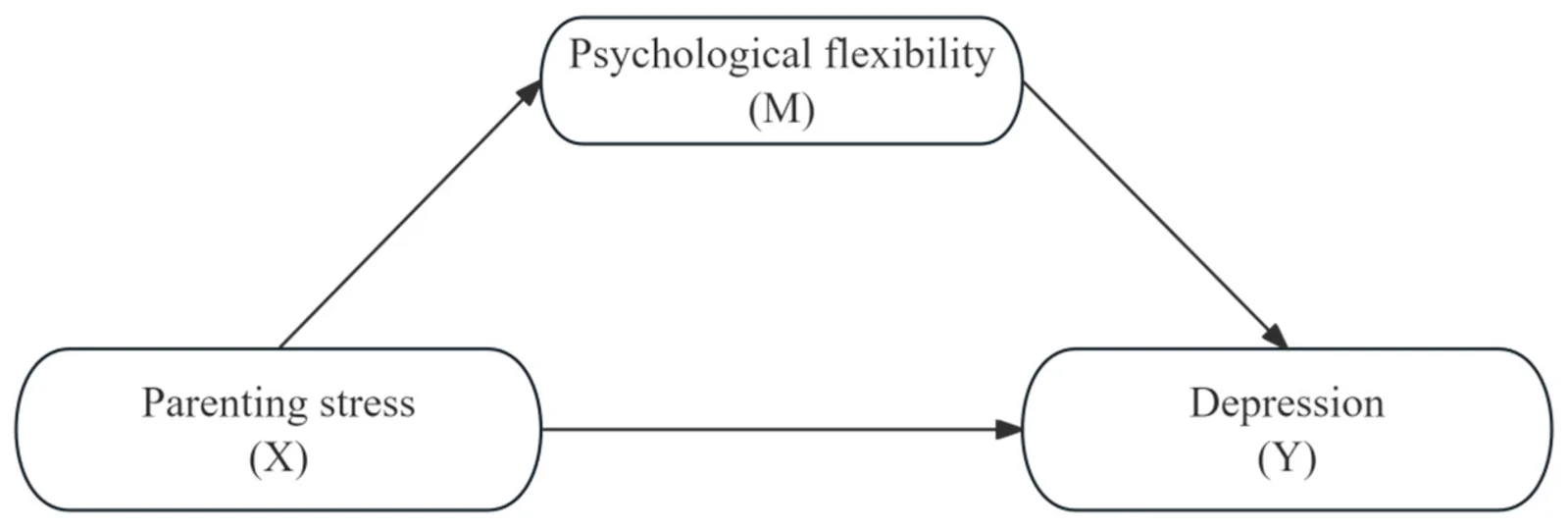
Conceptual diagram of the hypothesized partial mediation model (PROCESS Macro Model 4).

**H3.**  *Psychological flexibility acts as a mediator in the parenting stress–depression link. The directional pathway indicates that higher parenting stress is associated with reduced psychological flexibility, which in turn is associated with elevated depression (Figure 1).*

## 2. Methods

### 2.1. Participants and Procedure

The study adopted a cross-sectional survey design with convenience sampling to recruit participants from communities, maternal and child health centers, and kindergartens in Ya’an City, Sichuan Province, China. Data were collected from 9 October 2022 to 8 October 2025. (Data collection was conducted in multiple waves over three years to achieve the pre-estimated sample size of over 1000 participants. Importantly, the inclusion and exclusion criteria, questionnaire batteries, and survey procedures remained consistent throughout the entire study period.) Before recruitment, the research team contacted the relevant institutions (e.g., community service centers, maternal and child health hospitals) to obtain permission for conducting the survey. Trained research assistants distributed recruitment flyers and verbally introduced the purpose, content, and potential risks/benefits of the study to potential participants. Those who expressed interest in participating were invited to complete a preliminary screening to determine eligibility. Each participant completed the questionnaire once; no repeated measurements were taken.

Eligibility of participants was determined based on pre-specified inclusion and exclusion criteria. The inclusion criteria were (1) being the primary caregiver (defined as the individual who self-reported being most responsible for the child’s daily care and upbringing; this status was confirmed during on-site screening by trained research assistants) of an infant aged 0–36 months; (2) aged 18 years or older; (3) proficient in Mandarin (to ensure accurate understanding and completion of the questionnaire). The exclusion criteria were (1) self-reported diagnosis of severe psychiatric disorders (e.g., psychosis, bipolar disorder) or neurological disorders (confirmed by medical records if available); (2) infants with severe medical complications that required intensive care (e.g., severe congenital diseases, severe infections) during the survey period.

Before participation, all eligible participants were provided with a detailed information sheet explaining the study purpose, questionnaire content, anonymity guarantee, voluntary participation principle, and the right to withdraw from the study at any time without penalty. After fully understanding the study, participants who agreed to participate signed a written informed consent form. All questionnaires were paper-based, and participants completed them anonymously in a quiet environment (e.g., a separate room in the community service center or maternal and child health center) to avoid interference from others.

**Figure 2:**
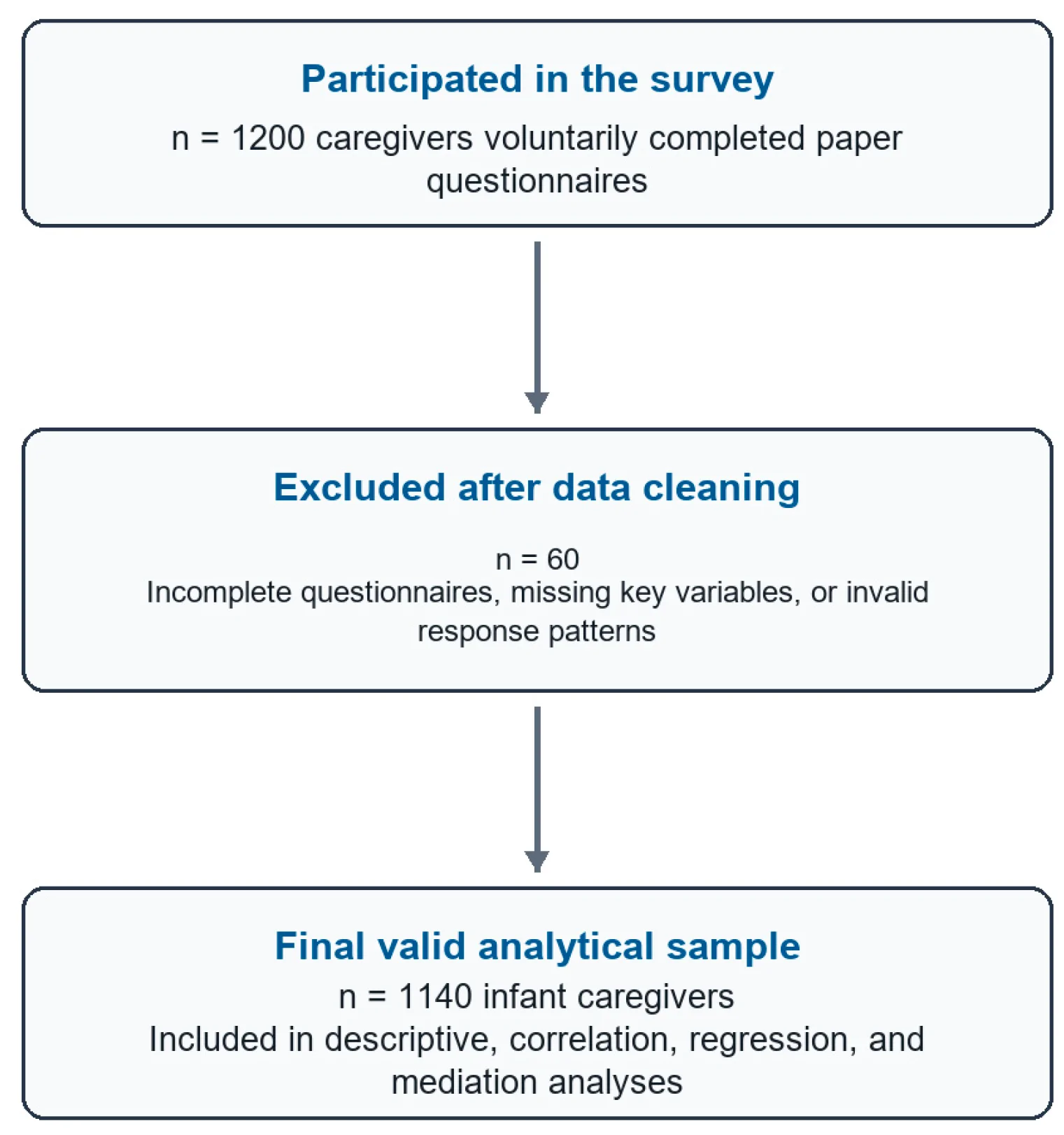
Participant flow diagram for recruitment, exclusions, and final analytical sample. The overall research protocol was obtained with ethical review approval by the Institutional Review Board of Stanford University (IRB Protocol #63680, Approval Date: 9 October 2022), which served as the approving ethics committee for this collaborative study. The study was implemented in China in collaboration with Southwestern University of Finance and Economics and with administrative permission from the participating community and maternal-child health service sites. All original data were collected locally in China, stored independently by the Chinese research team, and only aggregated, de-identified statistical results were shared with the overseas cooperative team. All procedures complied with ethical standards for human participant research.

Trained research assistants were on-site during the questionnaire completion process to answer any questions raised by participants and ensure that the questionnaires were filled out correctly. To avoid missing or invalid responses, research assistants checked each questionnaire immediately after completion. A total of 1200 caregivers voluntarily participated in the survey. Missing data and invalid responses were screened before analysis. Questionnaires were excluded if they lacked valid responses for any main scale (PSI-SF, AAQ-II, or PHQ-8), lacked required covariate information for the adjusted models, or showed invalid response patterns such as extensive missing items or logically inconsistent demographic information. 60 questionnaires (5.0%) were excluded, and the final valid analytical sample consisted of 1140 infant caregivers (95.0%) (Figure 2). Complete-case analysis was used; no item-level or scale-level imputation was performed. The initial sample size estimation was performed using G*Power 3.1 for a multiple regression model. Assuming a medium effect size (f^2^ = 0.15), a significance level of α = 0.05, and statistical power of 0.95 for a multiple regression model, the minimum required sample size was calculated as 160 [36]. The final valid sample (N = 1140) was considered sufficient for the planned regression-based mediation analysis.

### 2.2. Measures

#### 2.2.1. Demographic Characteristics

Participants reported demographic information, including caregiver gender, ethnicity, age, educational level, annual household income, as well as infant characteristics (gender, age in months, parity, delivery mode, and prematurity status). In this study, Parity refers to the total number of live births delivered by the biological mothers of the infants, including the target infants recruited in the sample.

#### 2.2.2. Parenting Stress

Parenting stress was assessed using the Chinese version of the Parenting Stress Index–Short Form (PSI-SF) [37]. The PSI-SF consists of 36 items across three subscales: Parenting Distress, Parent–Child Dysfunctional Interaction, and Difficult Child. Items are rated on a 5-point Likert scale ranging from 1 (strongly disagree) to 5 (strongly agree), with higher total scores indicating greater parenting stress. The Chinese version of the PSI-SF has demonstrated good reliability and validity in previous studies [38]. In the present study, Cronbach’s α = 0.92, indicating excellent internal consistency.

#### 2.2.3. Psychological Flexibility

The Acceptance and Action Questionnaire-II (AAQ-II) was used to assess experiential avoidance and psychological inflexibility. The original version was developed by Hayes et al. [39], and the revised version was proposed by Bond et al. to improve psychometric properties. [40]. The AAQ-II consists of 7 items rated on a 7-point Likert scale (1 = never true to 7 = always true), yielding total scores ranging from 7 to 49. Higher scores reflect greater experiential avoidance and lower psychological flexibility, that is, high psychological inflexibility [41]. In this study, Cronbach’s α = 0.90, indicating good internal consistency.

#### 2.2.4. Depression

Depression was assessed using the Chinese version of the Patient Health Questionnaire-8 (PHQ-8) [42]. The PHQ-8 measures the number of days in the past two weeks during which participants experienced eight core depression symptoms (e.g., anhedonia, depressed mood, sleep disturbance, fatigue, appetite changes, low self-worth, concentration difficulties, psychomotor changes). Each item is rated according to the number of days experienced (0–1 day, 2–6 days, 7–11 days, 12–14 days), scored from 0 to 3, resulting in total scores ranging from 0 to 24. A total score ≥ 10 indicates clinically significant depression [43,44]. In the current sample, Cronbach’s α = 0.79, indicating acceptable internal consistency.

### 2.3. Data Analysis

Data were analyzed using SPSS Statistics 27.0. Descriptive statistics were computed for demographic variables and study measures. Because PHQ-8 scores showed a marked floor effect [45], Spearman rank correlation analyses were used to examine bivariate associations among parenting stress, psychological flexibility, and depression. Multiple linear regression analysis was used to examine the associations between parenting stress and psychological flexibility on depression while controlling for demographic variables. To test the hypothesized mediating effect of psychological flexibility, mediation analysis was conducted using the PROCESS macro (Version 4.0) [46]. Indirect effects were estimated using bias-corrected bootstrapping with 5000 resamples and 95% confidence intervals (CIs). Mediation was considered significant if the 95% CI did not include zero. All statistical tests were two-tailed, and the significance level was set at α = 0.05.

## 3. Results

### 3.1. Sociodemographic Characteristics and Distribution of Depression

The sociodemographic characteristics of the participants and the distribution of depression are presented in Table 1. A total of 1140 primary caregivers of infants aged 0–36 months were included in the final analysis. The majority were female (*n* = 84.30%) and of Han ethnicity (75.18%). Most participants reported an annual household income exceeding 20,000 RMB (85.45%), and 45% had completed junior high school. The mean age of infants was 16.31 months (SD = 5.61). Among caregivers, 42.98% had one child, and 7.01% reported having a premature infant.

As shown in Table 1, the mean scores for the main study variables were as follows: parenting stress (M ± SD = 90.49 ± 16.26), psychological inflexibility (M ± SD = 18.94 ± 12.30), and depressive symptoms (M ± SD = 1.15 ± 2.24). The total PHQ-8 depression scores exhibited strong left skewness and an obvious floor effect in this sample. Using the standard clinical cutoff of PHQ-8 total score ≥ 10 to identify clinically meaningful depressive symptoms, we calculated the prevalence of elevated depression among infant primary caregivers as 3.16% (36 out of 1140 participants).

Group comparisons further indicated that depression differed significantly across annual household income (*p* = 0.001), educational attainment (*p* = 0.027), infant age (*p* = 0.012), and parity (*p* = 0.030). Specifically, caregivers with lower income and lower education levels reported higher levels of depression. Caregivers of younger infants (0–12 months) and those with only one child also exhibited significantly higher depression scores. No significant differences in depression were observed by caregiver gender, ethnicity, mode of delivery, infant gender, or prematurity status (all *p* > 0.05).

### 3.2. Correlations Among Study Variables

Spearman rank correlation analyses were conducted to examine associations among the main study variables (Table 2). Parenting stress was positively correlated with depression (r = 0.332, *p* < 0.01) and AAQ-II-measured psychological inflexibility (r = 0.425, *p* < 0.01). AAQ-II-measured psychological inflexibility was positively correlated with depression (r = 0.420, *p* < 0.01).

### 3.3. Multiple Linear Regression Analyses

Multiple linear regression analyses were performed to further examine the associations among the study variables after adjusting for annual household income, education level, and infant age (Table 3). Model 1 tested H1, Model 2 tested H2, and Model 3 examined the hypothesized mediation model proposed in H3.

In Model 1, after adjusting for annual household income, education level, and infant age, parenting stress was significantly associated with depression (*β* = 0.318, *t* = 11.462, *p* < 0.001). Annual household income (*β* = −0.091, *t* = −3.302, *p* < 0.01), education level (*β* = −0.061, *t* = −2.215, *p* < 0.05), and infant age (*β* = −0.069, *t* = −2.518, *p* < 0.05) were also significantly associated with depression. The model accounted for 13.0% of the variance in depression (*R*^2^ = 0.130, *R*^2^_adj_ = 0.127).

In Model 2, parenting stress was significantly associated with psychological inflexibility after adjustment for the same covariates (*β* = 0.394, *t* = 14.312, *p* < 0.001). The model accounted for 18.0% of the variance in psychological inflexibility (*R*^2^ = 0.180, *R*^2^_adj_ = 0.177).

In Model 3, after psychological inflexibility was entered into the adjusted model, parenting stress remained significantly associated with depression (*β* = 0.174, *t* = 6.142, *p* < 0.001). Psychological inflexibility was also significantly associated with depression (*β* = 0.357, *t* = 12.503, *p* < 0.001). Annual household income (*β* = −0.081, *t* = −3.246, *p* < 0.01) and infant age (*β* = −0.057, *t* = −2.334, *p* < 0.05) remained significantly associated with depression, whereas education level was no longer significant (*β* = −0.045, *t* = −1.817, *p* > 0.05). The model accounted for 23.8% of the variance in depression (*R*^2^ = 0.238, *R*^2^_adj_ = 0.235).

### 3.4. Mediation Analysis

To further examine the mediating role of psychological flexibility, a mediation analysis was conducted using the PROCESS Model 4. The Bootstrap method with 5000 resamples was applied to estimate the 95% confidence intervals.

As shown in Table 4, the indirect effect of parenting stress on depression through psychological flexibility was significant (*β* = 0.149, SE = 0.003, 95% CI [0.143, 0.155]). The indirect effect accounted for 45% of the total effect. The direct effect of parenting stress on depression remained significant (*β* = 0.186, SE = 0.004, 95% CI [0.178, 0.194]), indicating partial mediation (Figure 2). The total effect was significant (*β* = 0.335, SE = 0.004, 95% CI [0.327, 0.343]). Because the confidence intervals did not include zero, the mediating effect was statistically supported.

**Figure 3:**
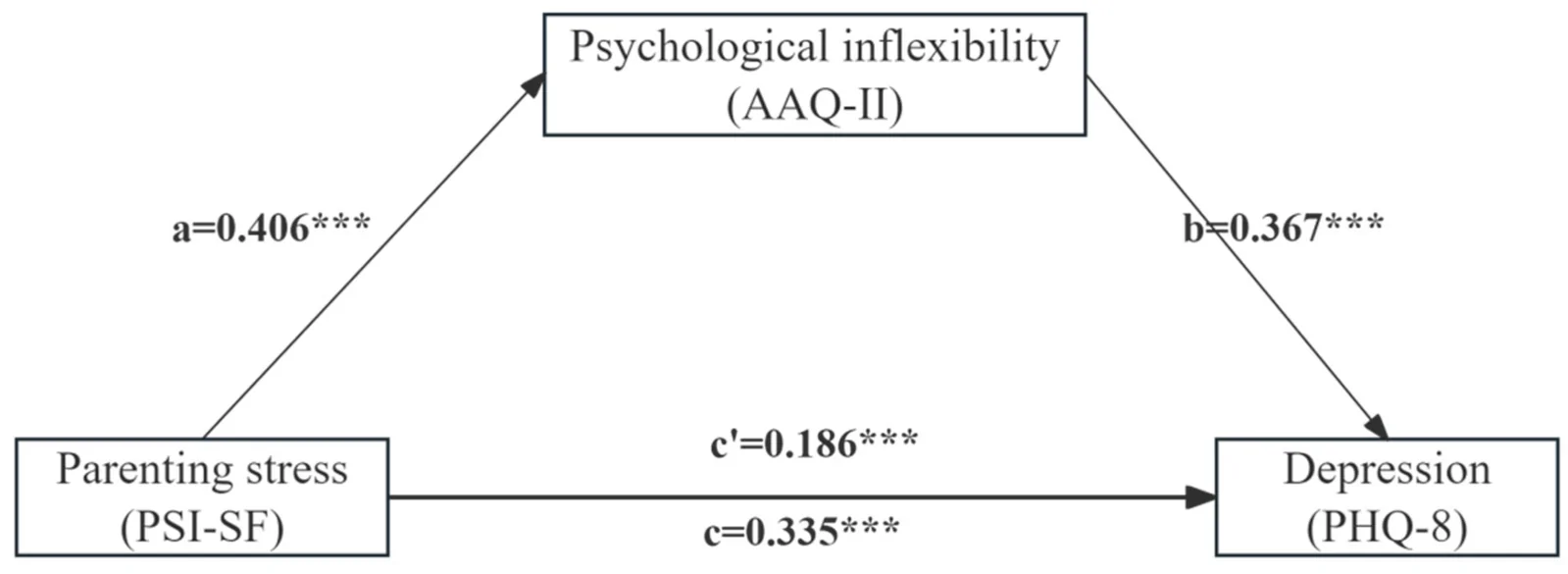
Standardized path diagram of the partial mediation model (PROCESS Model 4). Note: *** *p* < 0.001. Standardized beta path coefficients are displayed. Path a = parenting stress was associated with psychological inflexibility (AAQ-II); Path b = psychological inflexibility was associated with depression. Path c’ = residual direct effect of parenting stress on depression after controlling for the mediator; Path c = total effect of parenting stress on depression derived from the bivariate linear model. The indirect effect was calculated as a × b = 0.149.

The standardized mediation path coefficients are visualized in Figure 3. The total direct effect of parenting stress on depression was (*β* = 0.335) in the simple linear model (Model 1). After incorporating psychological flexibility (AAQ-II) as the mediating variable into Model 3, the residual direct effect of parenting stress on depression decreased to (*β* = 0.186). Parenting stress was positively associated with psychological inflexibility (*β* = 0.406), and higher psychological inflexibility was further associated with elevated depression (*β* = 0.367), consistent with the hypothesized mediating pathway rather than a univariate linear relationship.

## 4. Discussion

### 4.1. Main Findings

This study examined the mediating role of psychological flexibility between parenting stress and depression among Chinese primary caregivers of early childhood aged 0–36 months. Parenting stress was positively associated with depression, and psychological flexibility exerted a prominent partial mediating effect, accounting for 45% of the total effect. Higher parenting stress was associated with reduced psychological flexibility, which in turn was associated with elevated depression. These findings clarify the core psychological mechanism linking chronic caregiving burden to emotional dysfunction in infant caregivers.

The sample showed a low average PHQ-8 depression score (M ± SD = 1.15 ± 2.24), and a low prevalence of clinically elevated depression (3.16%), which is lower than the 10–32% range reported in many studies of Chinese infant and child caregivers [47,48]. This gap may be attributed to the fact that most participants reported minimal symptoms and participants’ stable household income and accessible intergenerational support [33,34,35]. Consistent with Chinese childcare norms, 84.3% of participants were female caregivers. Mothers bear the majority of round-the-clock infant care, leading to sleep deprivation, role overload, and work-family conflict, all robust risk factors for poor mental health [49,50]. Caregivers aged 26–40 years and infants under 12 months reported the highest depressive levels, consistent with evidence marking the first postpartum year as a high-vulnerability stage. Caregivers with higher educational attainment and higher household income reported lower depression scores. Caregivers with higher education levels and family incomes had lower depression scores, which is consistent with previous studies. [51].

Caregivers reported moderate-to-high parenting stress, driven by the intensive care demands of 0–36-month-old infants and rising work-family conflict for working women [6,7]. Consistent with stress and emotion regulation theory, chronic caregiving stress is associated with depletion of regulatory resources, accumulation of negative affect, and increased depression susceptibility [7,52,53]. This study provides empirical support for this theoretical pathway within Chinese infant caregiver populations.

The mediating effect of psychological flexibility between parenting stress and depression accounted for 45% of the total effect, and model comparison indicated that psychological flexibility was a core mediator, which was consistent with some previous studies [25,54]. In addition, the mediation results of this study support the cross-cultural universality of the ACT core framework. Despite the conflict between the individual value orientation of ACT and the family-centered collectivist norm, the path of “Parenting stress → psychological flexibility → depression” of Chinese caregivers remained stable. Culturally unique stressors such as intergenerational parenting conflict [20] are all associated with reduced psychological flexibility, providing evidence that this cross-diagnostic mechanism is not limited to Western samples.

### 4.2. Practical Implications

This study focused on the primary caregivers of infants and children aged 0–36 months and acknowledged their unique continuum of uncontrollable caregiving demands. By integrating multiple theoretical frameworks, this study strengthened the theoretical basis of the mediation pattern of the “parenting stress–psychological flexibility–depression” pathway, and thus tested the cross-cultural generalizability of the core transdiagnostic mechanism of ACT in the Chinese collective-style sample. To provide an empirical basis for the mental health intervention of early caregivers of cultural adaptability based on psychological flexibility training.

While reducing objective parenting stress remains an important goal, interventions that directly increase psychological flexibility may be a feasible and beneficial strategy that is associated with a lower risk of caregiver depression. ACT-based interventions targeting psychological flexibility are associated with reduced depression. ACT builds six core adaptive skills: acceptance, cognitive defusion, self-as-context, present-moment awareness, values clarification, and committed action [55]. However, longitudinal and experimental studies are needed to establish whether these associations reflect causal effects.

ACT-based support programs could be delivered through community parenting education programs, primary healthcare services, or digital mental health platforms [56]. In addition, integrating brief psychoeducation on psychological flexibility and simple mindfulness or acceptance-based exercises into routine postnatal care may help caregivers develop more adaptive coping strategies and reduce the risk of depression.

### 4.3. Limitations and Future Directions

This study only used a convenience sampling implementation study in China, which not only caused potential selection bias (for example, the PHQ-8 depression outcome variable showed a significant left bias and a significant floor effect in the results of this study), limiting national generalization and direct cross-cultural comparisons of ACT applicability. In addition, the aggregate data collection period was too long (2022–2025) to detect temporal fluctuations in core study variables. No multilevel screening was performed during recruitment, and the cross-sectional design of this study limits the ability to make causal inferences about the relationship between parenting stress, psychological flexibility, and depression. Variables such as social support, marital status, sleep quality, infant feeding patterns, personal history of depression, standardized measures of parent and infant temperament, employment status, caregiver status type, and relationship category (mother, father, grandparent, or other guardian) were not included in this study, which may be related to both independent and dependent variables in this study and may affect the stability and accuracy of the findings. Another limitation is that the a priori power analysis was based on a multiple regression framework rather than a mediation-specific Monte Carlo simulation for the indirect effect. Although the final sample size was large, future studies should use mediation-specific power estimation methods when mediation is the primary hypothesis.

In conclusion, future studies should appropriately design data collection to quantify and eliminate potential time bias. Stratified random sampling was used to improve the representativeness of the sample. To explore how cultural values modulate the strength of parental stress-recovery pathways to improve the cultural universality of ACT. Longitudinal studies are needed to elucidate temporal relationships between these variables and potential causal pathways, and randomized controlled trials are needed to directly test the effects of ACT interventions. The questionnaire should be extended to include all potential confounders, including more potential confounding variables, to further improve the scientificity and generalizability of the research, and provide more targeted mental health support strategies for the differences.

## 5. Conclusions

Depression among caregivers of early childhood can have significant consequences for both caregiver well-being and child development. The present study demonstrated that parenting stress is an important risk factor for depression and that psychological flexibility plays a significant partial mediating role in this relationship. Specifically, parenting stress was indirectly associated with depression through lower psychological flexibility. These findings contribute to a deeper understanding of the psychological mechanisms underlying caregiver depression and provide important implications for intervention development. Future prevention and intervention strategies may benefit from simultaneously reducing parenting stress and enhancing psychological flexibility, thereby supporting the mental health of caregivers and promoting the healthy development of infants.

## Figures and Tables

**Table 1:** Sociodemographic characteristics and depression scores (*n* = 1140).

Variable	*n* (%)/M ± SD	Depression (M ± SD)	*p* Value
**Gender**			0.07
Male	179 (15.70%)	1.05 ± 1.27	
Female	961 (84.30%)	3.25 ± 2.25	
**Ethnicity**			0.231
Han	857 (75.18%)	1.41 ± 2.44	
Others	283 (24.82%)	0.36 ± 1.09	
**Age (years)**			0.192
18–25	121 (10.61%)	1.03 ± 1.03	
26–40	650 (57.01%)	2.85 ± 2.14	
41–45	122 (10.70%)	1.25 ± 3.01	
46–59	219 (19.21%)	0.76 ± 1.48	
≥60	28 (2.45%)	0.65 ± 2.23	
**Annual household income (RMB)**			0.001
<8000	45 (3.94%)	2.65 ± 2.49	
8000–20,000	121 (10.61%)	2.98 ± 2.67	
20,001–50,000	370 (32.46%)	1.02 ± 2.14	
50,000–100,000	569 (49.91%)	0.85 ± 1.23	
>100,000	35 (3.07%)	0.48 ± 1.23	
**Education level**			0.027
Illiterate	40 (3.51%)	1.78 ± 3.45	
Primary school	152 (13.33%)	2.46 ± 1.69	
Junior high school	509 (44.65%)	2.15 ± 3.05	
High school	397 (34.82%)	1.75 ± 2.73	
College or above	42 (3.68%)	1.03 ± 2.43	
**Infant age (months)**			0.012
0–12	241 (21.14%)	2.85 ± 1.82	
13–24	809 (70.96%)	1.03 ± 1.34	
25–36	90 (7.89%)	1.05 ± 2.13	
**Parity**			0.030
1 child	490 (42.98%)	1.65 ± 2.08	
2 children	593 (52.02%)	0.95 ± 2.11	
≥3 children	57 (5.00%)	0.67 ± 1.27	
**Mode of delivery**			0.350
Vaginal delivery	547 (47.98%)	1.35 ± 2.51	
Cesarean section	593 (52.02%)	1.38 ± 2.23	
**Premature birth**			0.175
Yes	80 (7.01%)	1.75 ± 1.98	
No	1060(92.98%)	1.25 ± 2.67	
**Main study variables**			
Parenting stress	90.49 ± 16.26		
Psychological inflexibility	18.94 ± 12.30		
Depression	1.15 ± 2.24		

Note: *p* values based on Chi-square test for categorical variables and Mann–Whitney U or Kruskal–Wallis tests for non-normally distributed continuous variables. AAQ-II, Acceptance and Action Questionnaire-II. Parity = total number of live births delivered by the biological mothers of the infants, including the target infants recruited in the sample. PHQ-8 = 8-item Patient Health Questionnaire. Clinical depressive symptom threshold: PHQ-8 total score ≥ 10, prevalence = 3.16%. A prominent floor effect was observed for PHQ-8 scores, with most participants reporting minimal depressive symptoms.

**Table 2:** Spearman correlations among parenting stress, AAQ-II-measured psychological inflexibility, and depression (*n* = 1140).

Variable	1	2	3
**1. PSI-SF**	1		
**2. AAQ-II**	0.425 **	1	
**3. PHQ-8**	0.332 **	0.420 **	1

Note: ** *p* < 0.01. PSI-SF = Parenting Stress Index–Short Form; AAQ-II = Acceptance and Action Questionnaire-II; PHQ-8 = Patient Health Questionnaire-8. Spearman’s r values are reported as bivariate effect-size estimates.

**Table 3:** Multiple linear regression (*n* = 1140).

Variable	Model 1		Model 2		Model 3	
	*β*	*t*	*β*	*t*	*β*	*t*
Annual household income	−0.091	−3.302 **	−0.072	−2.641 **	−0.081	−3.246 **
Education level	−0.061	−2.215 *	−0.054	−1.982 *	−0.045	−1.817
Infant age	−0.069	−2.518 *	−0.038	−1.405	−0.057	−2.334 *
PSI-SF	0.318	11.462 ***	0.394	14.312 ***	0.174	6.142 ***
AAQ-II	-	-	-	-	0.357	12.503 ***
R^2^	0.130		0.180		0.238	
R^2^_adj_	0.127		0.177		0.235	
F	42.321 ***		62.309 ***		70.812 ***	

Note: * *p* < 0.05, ** *p* < 0.01, *** *p* < 0.001. PSI-SF = Parenting Stress Index–Short Form; AAQ-II = Acceptance and Action Questionnaire-II; PHQ-8 = Patient Health Questionnaire-8. Model 1 examined the association between parenting stress and depression after adjustment for annual household income, education level, and infant age. Model 2 examined the association between parenting stress and psychological inflexibility after adjustment for the same covariates. Model 3 examined the hypothesized mediation model after adjustment for the same covariates. Delta adjusted R^2^ = 0.108 comparing Model 3 with Model 1.

**Table 4:** Mediation analysis of psychological flexibility (*n* = 1140).

Effect	*β*	SE	95% CI	Proportion (%)
Indirect effect	0.149	0.003	0.143, 0.155	45%
Direct effect	0.186	0.004	0.178, 0.194	55%
Total effect	0.335	0.004	0.327, 0.343	100%

Note: *β* = standardized coefficient; SE = standard error; 95% CI = bootstrap 95% confidence interval. Indirect effect = mediating path via psychological inflexibility; Direct effect = direct effect of perceived stress on depressive symptoms; Proportion (%) = the relative contribution of each effect to the total effect. Non-zero 95% CI indicates a significant effect. All mediation estimates were adjusted for annual household income, education level, and infant age.

## Data Availability

The raw dataset generated and analysed during the current study cannot be publicly shared due to ethical restrictions and participant privacy protection. The study protocol was approved by the institutional ethics committee, and informed consent was obtained from all participants did not include provisions for open data sharing. Qualified reasonable requests for de-identified aggregated statistical results may be directed to the corresponding author.

## Public Involvement Statement

No public involvement activities were undertaken during any stage of this research. Caregivers of young children were recruited as research participants to complete questionnaires and semi-structured interviews for data collection only. They were not involved in the design of the study, development of data collection tools, data analysis, interpretation of findings, or preparation of this manuscript.

## Guidelines and Standards Statement

This manuscript was drafted in accordance with the Strengthening the Reporting of Observational Studies in Epidemiology (STROBE) Statement for cross-sectional observational studies. The STROBE checklist was used to guide the reporting of the study design, participants, variables, data sources, statistical methods, results, limitations, and interpretation.

## Use of Artificial Intelligence Statement

During the preparation of this manuscript, AI-assisted tools were only used for language translation and grammar polishing. Specifically, DeepL was used for translating non-English content into English, and Grammarly Premium was used to check and correct grammatical errors and sentence structures to improve the readability of the text. No AI tools were used for generating original text, analyzing research data, or drawing conclusions. All intellectual content, research design, and interpretation of the results were completed independently by the authors.
